# Removal of Mn, Fe, Ni and Cu Ions from Wastewater Using Cow Bone Charcoal

**DOI:** 10.3390/ma3010452

**Published:** 2010-01-14

**Authors:** Juan Carlos Moreno, Rigoberto Gómez, Liliana Giraldo

**Affiliations:** 1 Facultad de Ciencias, Departamento de Química, Grupo de Investigación en Sólidos Porosos y Calorimetría, Universidad de los Andes, Colombia; E-Mail: rgomez@uniandes.edu.co (R.G.); 2 Facultad de Ciencias, Departamento de Química, Universidad Nacional de Colombia, Bogotá, Colombia; E-Mail: lgiraldogu@unal.edu.co (L.G.)

**Keywords:** bone charcoal, immersion enthalpy, adsorption, heavy metals

## Abstract

Cow bone charcoal (CBC) was synthesized and used for the removal of metals ions (manganese, iron, nickel and copper) from aqueous solutions. Two different adsorption models were used for analyzing the data. Adsorption capacities were determined: copper ions exhibit the greatest adsorption on cow bone charcoal because of their size and pH conditions. Adsorption capacity varies as a function of pH. Adsorption isotherms from aqueous solution of heavy metals on CBC were determined. Adsorption isotherms are consistent with Langmuir´s adsorption model. Adsorbent quantity and immersion enthalpy were studied.

## 1. Introduction

At present, adsorption is widely accepted in environmental treatment applications throughout the world. Liquid–solid adsorption systems are based on the ability of certain solids to preferentially concentrate specific substances from solutions onto their surfaces. This principle can be used for the removal of pollutants, such as metal ions and organics, from wastewaters [1,2,3,4]. Extensive research has been carried out during the last ten years to find low-cost, high capacity adsorbents for the removal of metal ions. A wide range of adsorbents have been developed and tested, including several activated carbons [5,6,7,8]. A number of low-cost agricultural wastes; mud tire rubber and fly ash have been used for the removal of a range of metal ions. Other minerals and materials with potential for exchange sorption with cadmium, copper and zinc have been tested; among those are sodium calcium bentonite and bone char [9,10,11,12,13,14,15,16]. Several natural resources have also been studied, including tree fern, peat coal and chitosan.

Bone charcoal, a mixed adsorbent containing around 10% carbon and 90% calcium phosphate, is mainly produced by thermal treatment of bones. Structurally, calcium phosphate in bone charcoal is in the hydroxyapatite form [16]. Bone charcoal has traditionally been used in the sugar refining industry to remove color from sugar solutions. Recent studies have used bone charcoal to adsorb radioisotopes of antimony and europium ions from radioactive wastes [16,18,19,20,21,22]. Those authors suggested that chemisorption was the main operating mechanism for ^152^Eu^3+^ removal from the aqueous solution with a high degree of irreversible fixation on bone charcoal. They claimed that sorption is due to cation exchange of metal ions onto hydroxyapatite.

In the present work the adsorption of manganese, iron, nickel and copper ions from solutions, onto bone charcoal in agitated batch absorber vessels was studied. The main goal of this study is to examine the ability of bone charcoal to remove these ions from aqueous solution and therefore evaluate its potential to be used in wastewater treatment systems.

## 2. Experimental Section

### 2.1. Absorbent: Bone charcoal

Discarded cow bone residues from a cattle abatoir in Bogotá (Colombia) were tested. The bone charcoal (BC) residue is the result of a pyrolysis process according to the following conditions:

$$\text{Cow bones }\overset{800{}^{\circ}\text{C/5h}(\text{inert atmosphere})}{\to }\text{BC residue}+\text{steam}+\text{oil}+\text{ammonia liquor}$$

The BC residue was maintained under an inert atmosphere to avoid any oxidation. It was crushed and sieved to give uniform particle size (~ 40 mesh size) for use in the different applications. CBC was characterized by chemical analysis and the results shown in Table 1. Surface area and pore size distributions were determined from nitrogen adsorption-desorption isotherms obtained at 77 K with an automatic instrument (Quantachrome 3B). Samples were previously outgassed at 523 K for several hours. N_2_ adsorption data at relative pressures ranging from 10^−5^ to 0.99 were analyzed according to BET method for calculating apparent surface area S_BET_. The BET surface area of prepared cow bone charcoal was found to be 283 m^2^∙g^–1^. Pore volume was found to be 0.287 cm^3^·g^-1^.

**Table 1:** CBC physical and chemical properties.

Property	Value
Carbon content	11%
Hydrogen content	1.6%
Nitrogen content	4.3%
Chlorine content	2.1%
Ca_3_(PO_4_)_2_ content	77%
CaCO_3_ content	3.9%
Others (Mg, Fe, SiO_2_)	less than 1.0%
BET area	283 m^2^·g^−1^
Pore Volume, V_p_	0.287 cm^3^·g^−1^
Moisture	maximum 3%

Full-size table

### 2.2. Adsorbates: Metal ions

Analytical grade manganese(II) nitrate [Mn(NO_3_)_2_], iron(II) sulfate (FeSO_4_·7H_2_O), nickel(II) nitrate [Ni(NO_3_)_2_·6H_2_O], and copper(II) nitrate [Cu(NO_3_)_2_ ·5H_2_O] reagents from J.T. Baker were used in the experiments. Stock solutions of metal ions were prepared using deionized water. Metal ion solutions concentrations of were determined by atomic absorption spectrometry (AAS).

### 2.3. Adsorption equilibrium isotherm

Batch sorption experiments were conducted using 100 mL aliquots of pH adjusted test solutions containing 100 mg∙L^–1^ of each one of the ions Mn^2+^, Fe^2+^, Ni^2+^ and Cu^2+^ in monocomponent systems and placed in 250 mL amber closed bottles. A known quantity (0.01–0.15 g) of CBC was added to each bottle. Solutions were stirred at 200 rpm for periods ranging between 5 and 110 min at 298 ± 1 K. The bone charcoal was removed by filtration and the Mn^2+^, Fe^2+^, Ni^2+^ and Cu^2+^ molar concentrations were measured by atomic absorption spectroscopy (in a Perkin Elmer AAnalyst equipment) at the end of each time period. Blank solutions were also prepared and analyzed. Solution pH changes as metal ion concentration changes during the adsorption process. A previous survey was made to determine the solution pH which produces the maximum adsorption. The pH values of each metal ion solution were adjusted using either 0.01 N NaOH or 0.01 N HNO_3_ solutions and the volumes used were recorded to calculate the final solution volume.

### 2.4. Immersion enthalpy

Immersion enthalpies of CBC were determined in solutions of Mn^2+^, Fe^2+^, Ni^2+^ and Cu^2+^ with concentrations ranging from 20 to 100 mg∙L^−1^ for the maximum adsorption pH of 5.1. Immersion enthalpies were also determined for 100 mg∙L^−1^ solutions at all pH values studied. This determination was performed in a heat conduction microcalorimeter equipped with a stainless steel calorimetric cell [23]. Thirty mL of the solution to be used were pre-heated at 298 K, then placed in the cell. A sample of approximately 0.500 g CBC was weighed and placed inside the calorimetric cell in a glass ampoule. The microcalorimeter was then assembled. When the equipment reached the temperature of 298 K, potential readings were registered after a period of approximately 15 minutes, with readings every 20 seconds, the glass ampoule was broken and the generated thermal effect recorded. Electric potential readings were continue for approximately 15 minutes more and at the end of the experiment, the equipment was electrically calibrated.

## 3. Results and Discussion

### 3.1. Effect of cow bone charcoal dosage on adsorption

**Figure 1:**
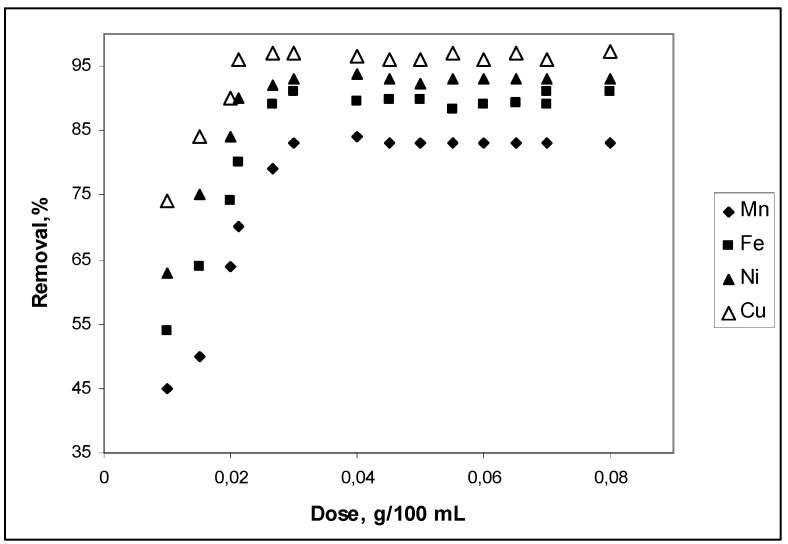
CBC adsorbent dosage effect on Mn^2+^, Fe^2+^, Ni^2+^ and Cu^2+^ removal. Conditions: C_o_, 20 mg·L^–1^; time of contact, 60 min; pH 5.1 and temperature, 298 K.

Figure 1 shows the removal of Mn^2+^, Fe^2+^, Ni^2+^ and Cu^2+^ from aqueous solutions of pH 5.1 as a function of added CBC. Cow bone charcoal dosage ranged from 0.01 to 0.08 g for the 100 mL of Mn^2+^, Fe^2+^, Ni^2+^and Cu^2+^ test solutions, equilibrated for 60 min. It can be seen that the maximum removal expressed as a percentage was between 75% and 98% from Mn to Cu at dosages between 0.02 g and 0.03 g of CBC. Ion removal increased quickly from 0.01 g to 0.02 g CBC dosages and reached a maximum for 0.03 g CBC. This fact may be associated with the M^2+^ ion availability at pH 5.1. From p*K*_h_ values, it can be concluded that, at pH 5.1, Mn^2+^ ions have a concentration 1,500 times greater than Cu^2+^ ions. On the other hand, hydrated Mn^2+^ ions have a volume almost 30% bigger than hydrated Cu^2+^ ions. Mn^2+^ ions are more likely to be in solution rather than adsorbed. The observed constancy in percentage ion removal beyond 0.03 g/100 mL may be an indicative of a very weak interaction between adsorbent and adsorbate. This interaction appears weaker with Mn^2+^ ions than with Cu^2+^ ions. Ion solution concentration seems to attain a steady state with adsorbed species, and so, no matter the quantity of adsorbent present, there will be a residual concentration of ions in solution. This fact determines a specific relation between ion concentration and adsorbent quantity.

Adsorption of metal ions on these types of materials is generally attributed to weak interactions between the adsorbents and adsorbates. Surface charges on substrates, as well as softness or hardness of the solutes are mostly responsible for the intensity of these interactions. Coulombic interactions can be observed for the ionic interexchange of cationic species with anionic sites in the materials and is determined by their surface areas.

### 3.2. Effect of contact time

**Figure 2:**
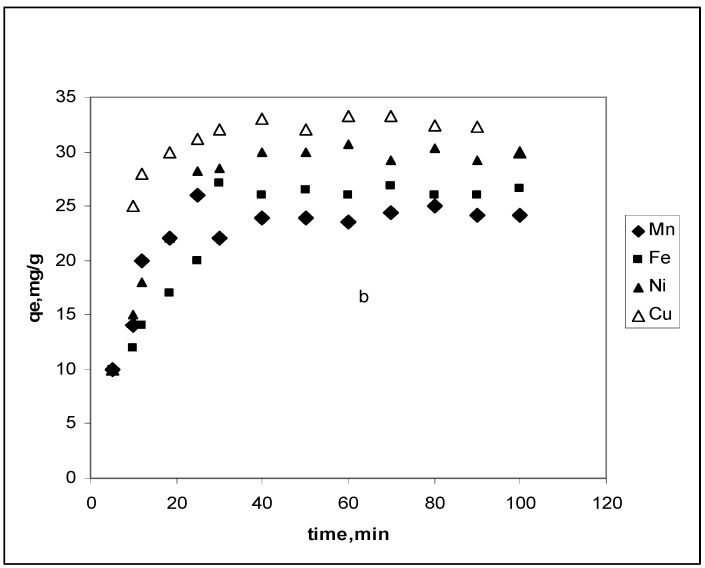
CBC contact time effect on Mn^2+^, Fe^2+^, Ni^2+^ and Cu^2+^ removal. Conditions: C_o_, 20 mg∙L^–1^; CBC dose, 0.02 g; pH 5.1 and temperature, 298 K.

Figure 2 shows contact time effect on the CBC removal of 20 mg∙L^–1^ Mn^2+^, Fe^2+^, Ni^2+^ and Cu^2+^. Removal increases with time and reaches a maximum after 20 min of agitation. Nevertheless, the order of affinity for the adsorbate is maintained: Mn^2+^ < Fe^2+^< Ni^2+^< Cu^2+^. This is associated with the size of the ion and the pore development in cow bone charcoal.

To analyze the sorption rates of Mn^2+^, Fe^2+^, Ni^2+^ and Cu^2+^ ions onto the CBC, two simple kinetic models were tested.

#### 3.2.1. Pseudo-first-order model

The pseudo-first order rate expression, popularly known as the Lagergren equation, is generally described by the following equation (Lagergren, 1898) [26]:

(1) $$\frac{dq}{dt}=k_{ad}\left(q_{e}-q\right)$$

where, q_e_ is the amount of the metal ions adsorbed at equilibrium per unit weight of sorbent (mg∙g^–1^); *q* is the amount of metal ions adsorbed at any time (mg∙g^–1^). Besides, *k*_ad_ is the rate constant min^–1^. Integrating and applying boundary conditions, *t* = 0 and *q_t_* = 0 to *t* = *t* and *q* = *q_t_*, Equation 1 takes the form:

(2) $$\ln\left(q_{e}-q_{t}\right)=\ln q_{e}-k_{ad}t$$

However, if the intercept does not equal the natural logarithm of the equilibrium uptake of metal ions, the reaction is not likely to be first-order, even if this plot has a high correlation coefficient with the experimental data [27]. Correlation coefficients were found to be between 0.9245 and 0.9865. The correlation coefficients are shown on Table 2, together with the Lagergren rate constants calculated from the slope of Equation 2 [26]. In order to obtain rate constants, the straight-line plots of ln (*q_e_* – *q_t_*) against *t* (time) were made (not shown here). This gave fairly straight lines for all four metal ions on the CBC. The intercept of this plot should give ln *q_e_*.

**Table 2:** Lagergren rate equation constants and pseudo second-order rate equation constants for Mn^2+^, Fe^2+^, Ni^2+^ and Cu^2+^ adsorption on CBC.

**Lagergren rate equations constants**			
**Metal ions**	***k_ad_* × min.**	***q*_e_ × (g·mg^–1^)**	**R^2^**
Mn^2+^	0.023	8.5	0.9443
Fe^2+^	0.028	9.3	0.9765
Ni^2+^	0.022	12.4	0.9865
Cu^2+^	0.018	15.8	0.9245
**Pseudo second-order rate equation constants**			
**Metal ions**	***h*_o_ × ( min·g·mg^–1^)**	***q*_e_ × (g·mg^–1^)**	**R^2^**
Mn^2+^	734.6	22.4	0.9991
Fe^2+^	1546.6	26.7	0.9993
Ni^2+^	1546.5	29.7	0.9999
Cu^2+^	1656.6	33.2	0.9999

#### 3.2.2. Pseudo-second-order model

The adsorption data was also analyzed in terms of a pseudo-second order mechanism given by [27]:

(3) $$\frac{dq}{dt}=k_{2}{\left(q_{e}-q_{t}\right)}^{2}$$

where, *k*_2_ is the rate constant (mg·g**^–1^**·min**^–1^**). Integrating the above equation and applying boundary conditions, *i.e.,* *t* = 0 for *q* = 0 and *t* = *t* for *q* = *q_t_*, gives:

(4a) $$\frac{t}{q_{t}}=\frac{1}{h_{\text{o}}}+\frac{1}{q_{e}}t$$

here, *h*_o_ is the initial adsorption rate. If the second-order kinetics is applicable, the plot of *t*/*q* against *t* in equation 4 should give a linear relationship from which the constants *q_e_* and *h*_o_ can be determined (plot not show here). Linear model gave a good fit to the experimental data. This means that the adsorption can be described by a pseudo-second order rate equation, hence *q_e_* and *h*_o_ were evaluated and presented in Table 2. R^2^ values are approximate the same for all four metal ions on CBC with a value of 0.9999. In the limit at initial adsorption time, *h*_o_ is defined as [28]:

(4b) $$h_{\text{o}}=k_{2}q_{e}^{2}$$

*k*_2_ was calculated for the four metal ions and are shown in Table 2. The results obtained are similar to a previous study (Horsfall, *et al.*, 2004) [28].

**Table 3:** Hydrolysis constants and ionic volumes of metals ions.

Heavy Metals Ions	Mn^2+^	Fe^2+^	Ni^2+^	Cu^2+^
**pK_h_**	10.7	10.1	9.40	7.53
**Hydrated volume (cm^3^·mol^–1^)**	189.6	174.5	147.8	147.8

M^2+^     + H_2_O   →   MOH^+^   + H^+^

MOH^+^ + H_2_O   →    M(OH)_2_ + H^+^

M^2+^     + 2H_2_O  →  M(OH)_2_ + 2H^+^

It can be seen that metal ions are easily adsorbed when the hydrated ionic size decreases. Ionic sizes of the heavy metals used change in the order of Mn^2+^ > Fe^2+^ > Ni^2+^ > Cu^2+^ (Table 3). Since CBC is used is a microporous adsorbent [21], metals penetrate easily into these pores when the ionic size becomes small [22]. According to the order above, Mn^2+^ must be the least adsorbed and Cu^2+^ must be the most easily adsorbed. This is compatible with our experimental results. On the other hand, high spin, transition metals complexes exhibits stabilities according to Irving-Williams series. Studied systems agree with this behavior suggesting that metal complex with the adsorbent may play an important role in adsorption processes.

### 3.3. Effect of pH

**Figure 3:**
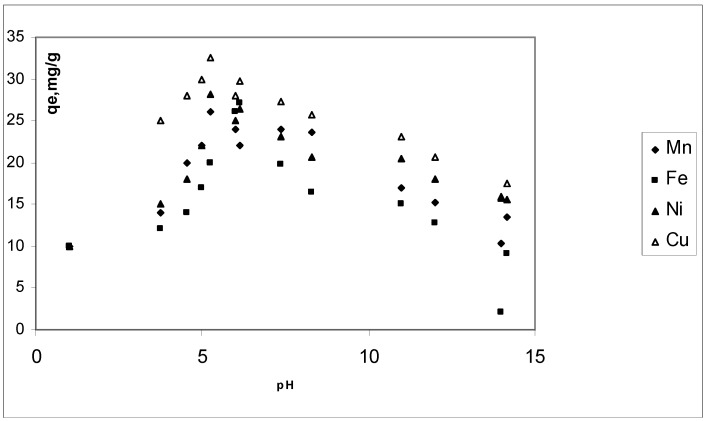
pH effect on CBC adsorption of Mn^2+^, Fe^2+^, Ni^2+^ and Cu^2+^. Conditions: C_0_, 20 mg∙L**^–1^**; CBC dose, 0.02 g; contact time, 20 min and temperature, 298 K.

Mn^2+^, Fe^2+^, Ni^2+^ and Cu^2+^ uptake as a function of hydrogen ion concentration was determined for pH values from 2 to 14. Below pH 5.1, hydrogen ions are likely to compete with manganese, iron, nickel and copper ions. At pH values above 8 manganese, iron, nickel and copper might precipitate as hidroxides. pH effects at equilibrium are presented in Figure 3. Maximum adsorption was observed about pH 5.1. In general, results indicated that the adsorption is highly pH dependant. Similar results have been reported in literature [20] S.K. Srivastava, R. Tyagi and N. Pant, Water Res. 23 (1989), pp. 1161–1165. Abstract | View Record in Scopus | Cited By in Scopus (106) [19].

pH values affect the species of heavy metals in aqueous solutions, and heavy metal removal increases as pH value rises, reaching a maximum around 5.1. Solution pH also affects the adsorbent and the surface charge of the CBC changes. Solubility product (K_sp_) calculations predict that the formation of Cu(OH)_2_, occurs at a pH value of 6. Precipitation occurs at pH 6, along with a *q*_e_ of 26.7 mg∙g^–1^. On the other hand, the *q*_e_ has a value of 35 mg∙g^–1^ when the initial pH was 5.1 (final pH of 2). This means that the removal of copper ions from the solution also contributes to the pH modification. However, at low initial pH values, below 4, the influence of adsorption is the only effect responsible for the reducing of copper ions in the solution. This suggests that the process is a suitable application for heavy metals removal because of its neutral and clean effluent.

### 3.4. Adsorption isotherms from aqueous solution

When the initial metal concentration rises, adsorption increases, while the binding sites are not saturated. Linear Langmuir isotherm allows the calculation of adsorption capacities and the Langmuir constants and is performed by the following equation:

(5) $$\frac{{\text{c}}_{eq}}{q}=\frac{1}{q_{\max}b}+\frac{{\text{c}}_{eq}}{q_{\max}}$$

Linear plots of c*_eq_*/*q* vs c*_eq_* (not shown), were used to calculate by means of linear regression equations, the parameters of the Langmuir isotherm. From these regression equations and the linear plots, the values of the Langmuir constants were calculated and are shown on Table 2. *q*_max_ and *b* were obtained from the slope and intercept of the plots. The essential characteristic of the Langmuir isotherms can be expressed in terms of a dimensionless constant separation factor or equilibrium parameter, R_L_, which is defined as [32]:

(6) $${\text{R}}_{L}=\frac{1}{\left(1+bc_{\text{o}}\right)}$$

where *b* is the Langmuir constant and c_o_ is the initial concentration of the metal ions. R_L_ value indicates the shape of the isotherm. R_L_ values between 0 and 1 indicate favorable absorption [33]. R_L_ equal to 0 indicate irreversible absorption, R_L_ = 1 is linear and R_L_ > 1 is unfavorable. From our study, R_L_ values for Mn^2+^, Fe^2+^, Ni^2+^ and Cu^2+^ ions adsorption ranged from 0.0050 to 0.0060. This, for an initial metal ions concentration of 600 mg∙L**^–1^**, therefore, the adsorption process is favorable.

The Freundlich isotherm was chosen to estimate the adsorption intensity of the adsorbent towards the adsorbate. It is represented by the equation [34]:

(7) $$q=K_{\text{F}}{\text{c}}_{eq}^{\frac{1}{n}}$$

where c*_eq_* is the equilibrium concentration (mg∙L^–1^), *q* is the ion amount adsorbed (mg∙g^–^^1^) and *K*_F_ and *n* are constants incorporating all parameters affecting the adsorption process, such as adsorption capacity and intensity respectively. Linear form of Freundlich adsorption isotherm was used to evaluate the sorption data and is represented as [34]:

(8) $$\ln q=\ln K_{\text{F}}+\frac{1}{n}\ln{\text{c}}_{eq}$$

The linear regression equation for the Freundlich adsorption isotherm is shown on Table 4. Values of K_F_ and *n* were calculated from the intercepts and slopes of the Freundlich plots respectively and are shown on this table. Adsorption is favorable for values 0.1 < 1/n < 1.0 [35].

**Table 4:** Isotherm parameters of Mn^2+^, Fe^2+^,Ni^2+^ and Cu^2^ adsorption on cow bone charcoal.

		Freundlich model			Langmuir model			
Metal	Linear K_D_ (L/g)	K_F_	1/*n*	R^2^	*q*_max_ (mg/g)	b (L/g)	R_L_	R^2^
Mn^2+^	6.76	14.457	0.315	0.9587	29.56	1.12	0.006	0.9987
Fe^2+^	6.99	23.545	0.425	0.9643	31.43	1.18	0.005	0.9988
Ni^2+^	7.89	26.876	0.643	0.9745	32.54	1.25	0.005	0.9988
Cu^2+^	8.88	34.865	0.759	0.9876	35.44	1.34	0.005	0.9999

**Figure 4:**
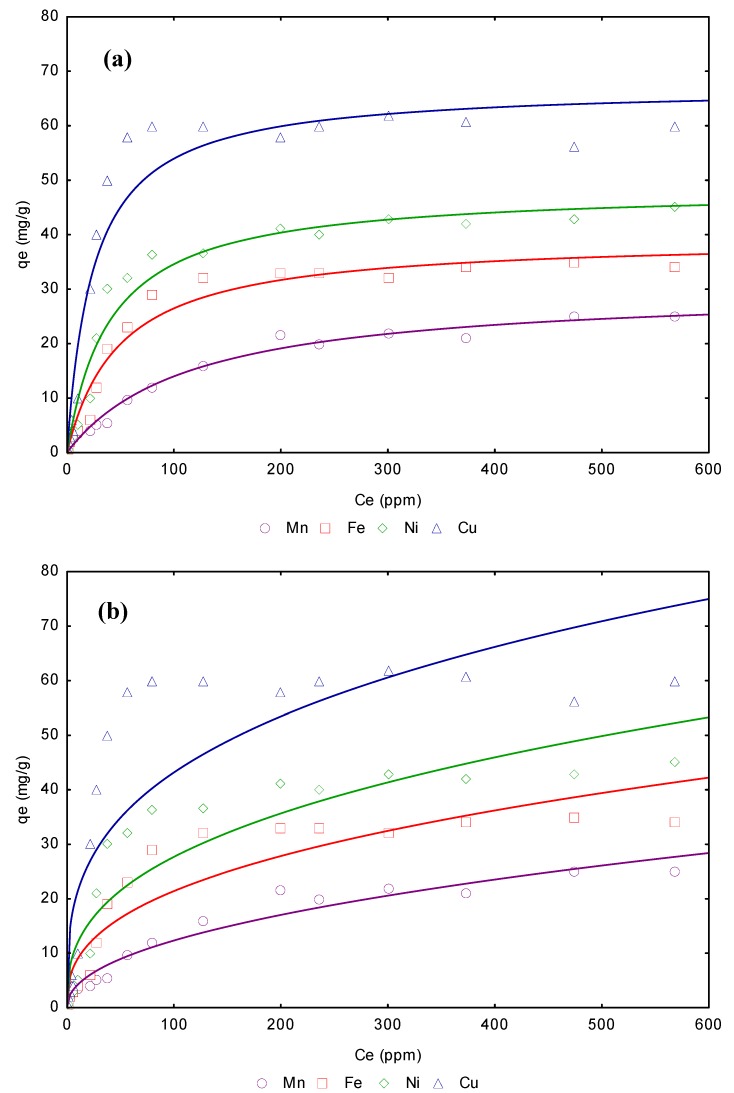
(a). CBC adsorption isotherms removal of Mn^2+^, Fe^2+^,Ni^2+^ and Cu^2+^ from aqueous solution, Langmuir model. (b). CBC adsorption isotherms removal of Mn^2+^, Fe^2+^,Ni^2+^ and Cu^2+^ from aqueous solution, Freundlich model.

The Freundlich equation frequently gives an adequate description of adsorption data over a restricted range of concentration, even though it is not based on any theoretical background. Apart from a homogeneous surface, the Freundlich equation is also suitable for a highly heterogeneous surface and an adsorption isotherm lacking a plateau, indicating a multi-layer adsorption [36]. Values of 1/n, less than unity are an indication that significant adsorption takes place at low concentration but the increase in the amount adsorbed with concentration becomes less significant at higher concentration and vice versa [37]. The magnitude of K_F_ and *n*, shows that it is possible an easy separation of heavy metal ion from aqueous solution and a high adsorption capacity. Also, as the K_F_ value increases, the greater the adsorption intensity. Therefore, the higher K_F_ values for Cu^2+^ confirms by these model that the adsorption capacity of is greater than that of the others ions. On the other hand, a relatively high R^2^ value indicates that this model is adjusted more confidently; this parameter is shown in the Table 4. According to the obtained values, the Langmuir model fits better the experimental data of the present study. Figure 4a and b shows adsorption isotherms related to Mn^2+^, Fe^2+^, Ni^2+^ and Cu^2+^ adsorption from aqueous solution on CBC. Continuous lines represent the non-linear regression adjustment of these isotherms fitted the Freundlich adsorption isotherm model and Langmuir isotherm model.

### 3.5. Immersion enthalpies

**Figure 5:**
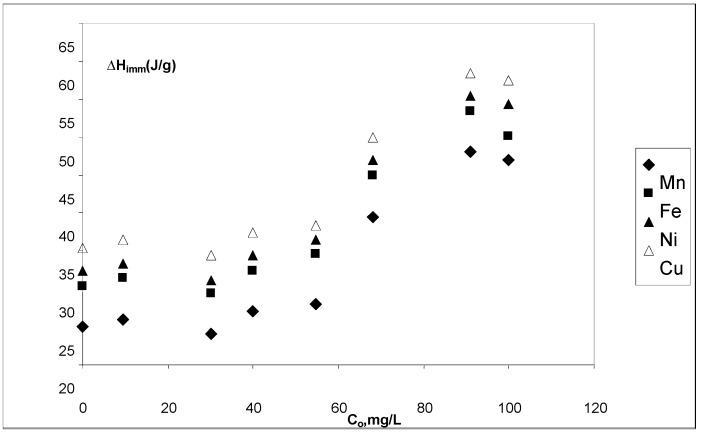
Immersion enthalpies for Mn^2+^, Fe^2+^, Ni^2+^ and Cu^2+^ aqueous solutions ions concentration at pH 5.1. T = 298 K.

Results show that immersion enthalpies are constant at low initial concentrations. Initial concentrations above 40 mg∙L^–1^ exhibited a steady increase up to 90 mg∙L^–1^. The highest value of enthalpy was obtained for the immersion of cow bone charcoal in the copper ions solutions, while the lower value of immersion enthalpy was obtained for the immersion of cow bone charcoal in the solutions of manganese. Enthalpy values were between –60 J∙g^–1^ (Cu^2+^–CBC) and –45 J∙g^–1^ (Mn^2+^–CBC), as shown in Figure 5. This behavior agrees with textural characteristics of cow bone charcoal and the sizes of the ions under study. It should be noted that the behavior of immersion enthalpies in the solid prepared in this work, is very similar to that of an isotherm.

### 3.6. Removal of Mn^2+^, Fe^2+^, Ni^2+^ and Cu^2+^ from wastewater

As an approximation of the results of the present work for application to a real problem, we proposed use an industrial wastewater sample. For that purpose we chose waste from a textile industry for which the content of studied metals was determined. The sample was carefully treated with the aim of performing an analysis of each one of the ions of interest and we evaluated the adsorption capacity as the sole comparison parameter. They are analyzed one by one in order to avoid multicomponent system generation which could produce bias in the obtained results.

Wastewater samples collected in our research laboratory from a textile industry were found to contain more of 500 mg∙L**^–1^** of Mn^2+^, Fe^2+^, Ni^2+^ and Cu^2+^, among other organic and inorganic components. Six samples were treated with nitric acid, followed by pH adjustment and sorption with CBC under optimized conditions described before. Metal ions were analyzed one at a time by atomic absorption spectrometry, using a complexing agent to avoid interference of ions different from that analyzed. Assay for manganese, iron, nickel and copper in the final effluents indicates 75.0% maximum removal of the ions originally present in the samples. The minimum removal was 53% for Mn(II). Mean standard deviation was 1.0%. These results show that CBC is an suitable material for use in the removal of these ions. However these findings should be analyzed carefully because of, in spite of procedures employed in order to avoid interferences in the assays, the sample complexity does not allow us to assure confidence in the results.

### 3.7. Mechanism of adsorption

Although cow bone charcoal displays relative low surface area (283 m^2^∙g^–1^), it shows high copper removal capacity (34.9 mg∙g^–1^). Cow bone charcoal analysis indicates that it consists of calcium phosphate as a major component (Table 1). It has been demonstrated that calcium phosphate acts not only as a source of adsorption centers but also enables ion-exchange process [24,25]:

≡PO^−^ + H^+^ → ≡POH

≡CaOH_2_^+^   → ≡CaOH + H^+^

In the presence of Cu^2+^, the following reactions may occur:

≡POH + Cu^2+^   → ≡POCu^+^ + H^+^

≡PO^−^ + Cu^2+^     → ≡POCu^+^

≡CaOH + Cu^2+^ → ≡CaOCu^+^ + H^+^

This mechanism is similar to that previously suggested for the sorption of Zn^2+^ and Ca^2+^ on calcium phosphate [24].

## 4. Conclusions

From the experiments, it can be concluded that the CBC has the ability to retain Mn^2+^, Fe^2+^, Ni^2+^ and Cu^2^ metals ions from aqueous solutions at the studied concentrations. Removal of heavy metals (manganese, iron, nickel and copper) from aqueous solution was possible using a activated carbon obtained from cow bone (CBC). It was seen that adsorption took place for the four metals within 25 minutes for the concentration levels studied. Under our experimental conditions and for the studied heavy metals pH plays an important role in the adsorption process, particularly on the adsorption capacity. The pH selected for an optimal rate of adsorption for all ions investigated is 5.1. It is shown that CBC has a relatively high adsorption capacity for these heavy metals; the quantities adsorbed per gram of CBC at equilibrium (*q_e_*) are 29.56 mg∙g^–^^1^ for Mn^2+^, 31.43 mg∙g^–^^1^ for Fe^2+^, 32.54 mg∙g^–^^1^ for Ni^2+^ and 35.44 mg∙g^–^^1^ for Cu^2+^. This adsorption is described by an isotherm of type I and is fully matched by the Langmuir isotherm. The kinetics of the manganese, iron, nickel and copper adsorption on the CBC was found to follow a pseudo-second-order rate equation. This method has an additional advantage, as it could be applied in developing countries due to the low cost.
